# Laser-Based Monitoring of CH_4_, CO_2_, NH_3_, and H_2_S in Animal Farming—System Characterization and Initial Demonstration

**DOI:** 10.3390/s18020529

**Published:** 2018-02-09

**Authors:** Dorota Stachowiak, Piotr Jaworski, Paweł Krzaczek, Grzegorz Maj, Michał Nikodem

**Affiliations:** 1 Laser Sensing Laboratory, Wroclaw Research Centre EIT+, Stabłowicka 147, 54-066 Wrocław, Poland; dorota.stachowiak@eitplus.pl (D.S.); piotr.jaworski@eitplus.pl (P.J.); 2 Department of Power Engineering and Transportation, Faculty of Production Engineering, University of Life Sciences in Lublin, Głęboka 28, 20-612 Lublin, Poland; pawel.krzaczek@up.lublin.pl (P.K.); grzegorz.maj@up.lublin.pl (G.M.); 3 Department of Optics and Photonics, Faculty of Fundamental Problems of Technology, Wrocław University of Science and Technology, Wybrzeże Wyspianskiego 27, 50-370 Wrocław, Poland

**Keywords:** laser spectroscopy, wavelength modulation spectroscopy, methane, ammonia, hydrogen sulfide

## Abstract

In this paper, we present a system for sequential detection of multiple gases using laser-based wavelength modulation spectroscopy (WMS) method combined with a Herriot-type multi-pass cell. Concentration of hydrogen sulfide (H_2_S), methane (CH_4_), carbon dioxide (CO_2_), and ammonia (NH_3_) are retrieved using three distributed feedback laser diodes operating at 1574.5 nm (H_2_S and CO_2_), 1651 nm (CH_4_), and 1531 nm (NH_3_). Careful adjustment of system parameters allows for H_2_S sensing at single parts-per-million by volume (ppmv) level with strongly reduced interference from adjacent CO_2_ transitions even at atmospheric pressure. System characterization in laboratory conditions is presented and the results from initial tests in real-world application are demonstrated.

## 1. Introduction

Monitoring gas emissions has become an important issue in the livestock sector [1,2,3,4]. Quality of air affects animals, has an impact on people who live nearby (due to odors) and climate (due to greenhouses gases emission). The environmental impact of pig farming (pig sector is the biggest contributor to global meat production) on air quality primarily includes emission of methane, ammonia, and hydrogen sulfide [5,6,7,8]. Proper assessment of emission rates requires all these gases to be detected in the continuous manner, at low cost, and with sensitivity and accuracy at single ppmv level. The existence of this problem was confirmed by the preliminary tests carried out in research facilities using portable, handheld devices or gas sampling bags for subsequent laboratory analysis. Fortunately, these requirements can be achieved when laser spectroscopy is applied. 

Laser spectroscopy is a powerful tool in chemical analysis. When implemented in the infrared spectral region, it can provide high sensitivity, high selectivity, robustness, and fast acquisition times. The strongest fundamental molecular transitions are located in the mid-infrared (beyond 3 µm) allowing for trace-gas detection down to or even below ppbv and pptv levels [9,10,11,12,13,14,15,16]. The near-infrared region offers overtone transitions that are usually from one to three orders of magnitude weaker. However, when detection at ppmv levels is needed, it is still a very attractive alternative, as it provides relatively cheap light sources and detectors (both can be operated at room temperature) and often benefits from using optical fiber-based components (fiber-coupled laser diodes and photodetectors, couplers, isolators, etc.) [17,18,19]. Near-infrared sources are frequently combined with a wavelength modulation spectroscopy (WMS) and multi-pass cells to provide sensitivities at or below ppmv levels for various species, including methane [20], ammonia [21], hydrogen sulfide [22], and carbon dioxide [23]. Simple and robust systems can fill the gap between relatively expensive instruments based on mid-infrared quantum cascade lasers (which can be few orders of magnitude more sensitive that required in our target application) [13,24] and portable, handheld devices which are cheap, but not sensitive enough (e.g., instruments for methane sensing are designed to detect it starting from relatively high levels such as 0.1%). 

In this paper, we present a laser-based system operating in the near-infrared spectral region that allows for CH_4_, NH_3_, and H_2_S detection at single-ppmv levels using three fiber-coupled laser diodes operated at 1651, 1531, and 1575 nm. Additionally, a laser diode at 1575 nm enables CO_2_ monitoring. The system is characterized in laboratory conditions and initial results of the first field tests in a pig farm are demonstrated.

## 2. System Design

**Figure 1:**
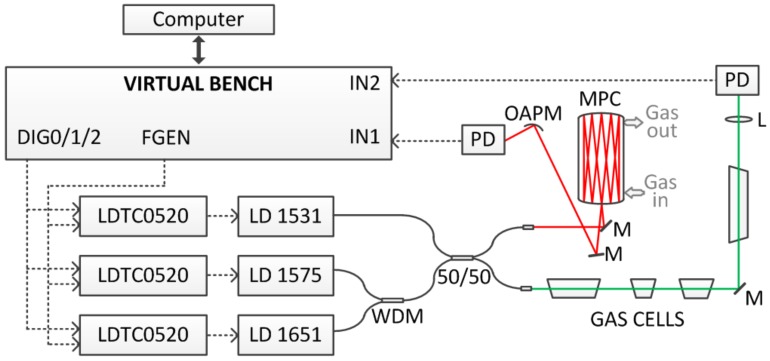
Schematic diagram of the system: FGEN—function generator; DIG0/1/2—digital outputs; LDTC0502—laser current driver with temperature controller (from Wavelength Electronics); WDM—1650nm/1550nm coupler; 50/50—broadband coupler; M—mirror, L—lens; OAPM—off-axis parabolic mirror; PD—photodetector; MPC—multi pass cell.

**Figure 2:**
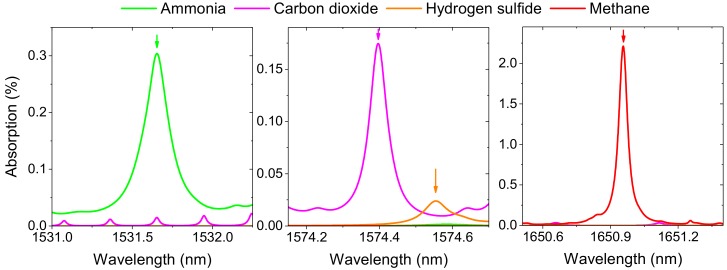
HITRAN-based simulation of 20 ppmv of methane, 10 ppmv of ammonia, 1000 ppmv of carbon dioxide, and 5 ppmv of hydrogen sulfide (these are typical concentrations that we expect to find in the field works). Target transitions are indicated with arrows.

Figure 1 shows the schematic diagram of the system whereas details on chosen spectral regions and target transitions are provided in Figure 2 and Table 1 (required detection limits were defined based on expected changes of concentrations and preliminary tests using gas sampling bags and laboratory analysis). Three laser diodes (Gooch & Housego, Ilminster, UK, model AA1401 at 1531 nm, NTT Electronics, model NLK1L5EAAA at 1575 nm and NTT Electronics, model NLK1U5EAAA at 1651 nm) were combined using fiber couplers and sent into two sensing paths. The first was a measurement path (shown in red), with a multi-pass cell, an off-axis parabolic mirror and a photodetector (Thorlabs, Newton, NJ, USA, PDA10CS-EC). The multi-pass cell was built using two spherical mirrors (Thorlabs, CM508-200) placed 397 mm away from each other. Approximately 30 m path length with volume of less than 800 mL was obtained using a Herriott type design which is relatively stable to small perturbations (e.g., due to temperature variations). The second path was a calibration section (shown in green) with four gas cells, a lens, and a photodetector (Thorlabs, PDA10CS-EC). Gas cells were: 5% NH_3_ in air (100 mm length), pure H_2_S (50 mm length), 4% CH_4_ in nitrogen (25 mm length), and 32% CO_2_ in air (150 mm), all at atmospheric pressures. The same gas cells were also used in the system characterization described in the following section. Gas cells with methane and hydrogen sulfide were provided by Wavelength References (Corvallis, OR, USA). Cells containing ammonia and hydrogen sulfide were made in-house and concentrations were determined through recording of target absorption line and spectral fitting using HITRAN database. Signals from both photodetectors were fed into the input channels of a Virtual Bench device (from National Instruments, Austin, TX, USA). Data acquisition was synchronized with a function generator providing sinusoidal wavelength modulation (*f*_m_ = 2.5 kHz) to the selected laser diode allowing for a WMS-based measurements [20,21,22,23,25,26,27,28,29,30,31,32,33,34]. For laser diodes at 1651 nm and 1531 nm we have selected modulation depth that was maximizing 2*f* WMS signals. For the laser diode at 1575 nm, modulation depth was adjusted to provide the smallest cross interference between spectral features of H_2_S and CO_2_ in 4*f* WMS spectrum. Digital outputs of Virtual Bench could be used to turn on/off laser diodes for sequential detection of CH_4_, CO_2_/H_2_S and NH_3_. Acquired signal was processed using a LabVIEW program. It allowed for lock-in detection of selected harmonic signals in a line-locked mode, when laser wavelength was adjusted to the transition center, or in a spectral scan mode, with the wavelength being scanned through changes of the injection current for full WMS spectrum recording and analyzing. WMS amplitude at 1 × *f*_m_ was also recorded for power normalization purposes [23,26,27].

### 2.1. Methane and Ammonia Detection

**Figure 3:**
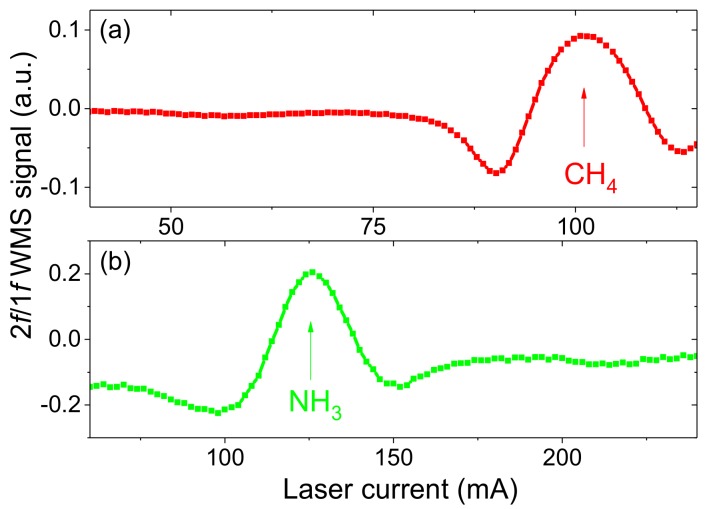
Recorded 2*f*/1*f* WMS spectra of methane (**a**) and ammonia (**b**) samples.

Figure 3 shows 2*f*/1*f* WMS spectra of methane and ammonia samples inside glass cells. Single spectral features are present, free from interference of other gases and with small, relatively flat baseline.

### 2.2. Carbon Dioxide and Hydrogen Sulfide Detection

**Figure 4:**
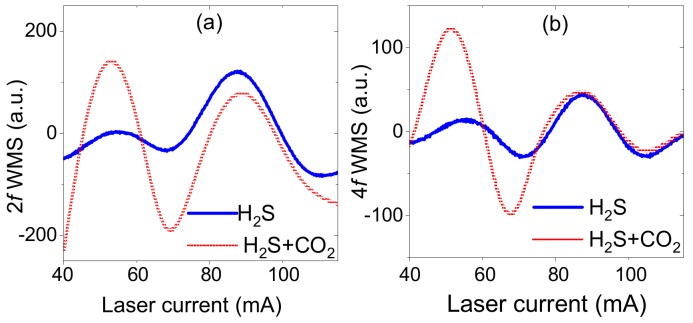
Recorded 2*f* (**a**) and 4*f* (**b**) WMS spectra of pure hydrogen sulfide (blue) and hydrogen sulfide with a presence of carbon dioxide (red). With 4*f* WMS detection it is much simpler to separate two main spectral features and baseline which greatly simplifies further signal analysis of weaker H_2_S line.

Detecting CO_2_ and H_2_S at 1574.5 nm is more challenging. When CO_2_ concentration is above 300 ppmv and H_2_S concentration is at single ppmv level, the hydrogen sulfide line is surrounded by stronger features from carbon dioxide transitions [22,35]. This is demonstrated in Figure 4a where two 2*f* WMS spectra are presented. The first was recorded with H_2_S sample only (pure H_2_S at 50 mm which corresponds to 5 × 10^4^ ppmv × m). The second was measured after setup was additionally filled with carbon dioxide, resulting in CO_2_ concentration of approximately 50% (with path-length of 30 m; this is more than two orders of magnitude times higher concentration than the concentration of H_2_S). It is clearly visible that the signal from hydrogen sulfide (both baseline and its amplitude at the line center) is affected by the wings of CO_2_ lines. In these conditions H_2_S concentration retrieval is still possible, but it becomes challenging and prone to errors. This issue can be addressed with analysis of WMS signal at higher harmonics of the modulation frequency [31]. In the presented system detection of 4th harmonic was implemented. The 4*f*/1*f* WMS signal typically has smaller baseline. Moreover, all spectral features recorded at higher harmonics become narrower (comparing to 2*f* WMS spectra), therefore cross-interference between neighboring transitions can be reduced [29,33,34]. This is demonstrated in Figure 4b. After filling the system with carbon dioxide spectral feature of hydrogen sulfide remains almost unchanged. Only small changes in the wings are observed which do not affect baseline or signal amplitude at the transition center. This reduced interference from CO_2_ transitions requires using 4*f*/1*f* detection with non-optimal wavelength modulation amplitude but it simplifies concentration retrieval and makes it more accurate. At the same time, 2*f*/1*f* detection can be still used for CO_2_ detection. 

## 3. System Characterization

### 3.1. Minimum Detection Limits

**Figure 5:**
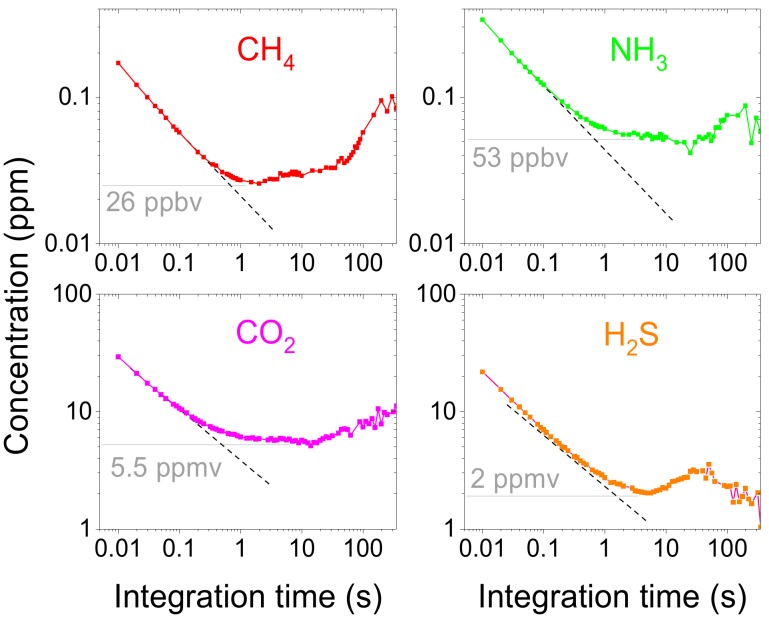
The Allan deviation plots measured for the system operated in a line-locked mode (i.e., with a laser wavelength parked at the transition center). Detection limits are calculated for a path length of 30 m.

For minimum detection limit (MDL) characterization each cell with gas under study was placed in line with a multi-pass cell, laser wavelength was adjusted to the transition center and WMS amplitude was recorded for subsequent Allan deviation analysis [36]. This configuration (gas cell in line with multi-pass cell) guarantees that any drifts that result from using multi-pass cell (e.g., from path length fluctuations, fringes etc.) will be visible in Allan deviations. At the same time, multi-pass cell was sealed tightly in order to minimize the impact of any changes in ambient concentrations of measured gases. For methane, ammonia, and carbon dioxide 2*f*/1*f* WMS amplitude was recorded, whereas 4*f*/1*f* WMS amplitude was measured for hydrogen sulfide sample. Figure 5 shows Allan deviation for each gas (detection limit was calculated assuming path length of 30 m). In all cases, the minimum is reached for integration time of ~5 s (this is obtained without active wavelength stabilization). For ammonia and hydrogen sulfide measurements, Allan deviation stays flat even for integration times longer than 100 s. In the case of methane and carbon dioxide, some drifts are observed which are most likely due to changes in ambient CH_4_ and CO_2_ concentrations inside multi-pass cell. Obtained MDLs are: 26 ppbv for CH_4_ (840 ppbv × m × Hz^−1/2^), 53 ppbv for NH_3_ (1.8 ppmv × m × Hz^−1/2^), 5.5 ppmv for CO_2_ (180 ppmv × m × Hz^−1/2^), and 2 ppmv for H_2_S (82.5 ppmv × m × Hz^−1/2^). These MDLs correspond to fractional absorptions of 2.9 × 10^−5^ (methane), 1.7 × 10^−5^ (ammonia), 0.6 × 10^−5^ (carbon dioxide), and 9.9 × 10^−5^ (hydrogen sulfide).

**Figure 6:**
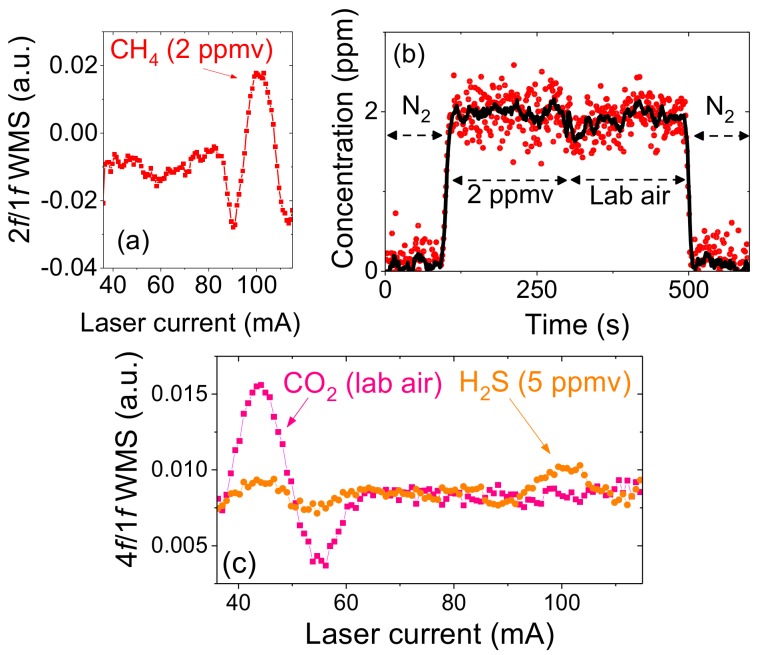
(**a**) Spectrum of methane at 1651 nm recorded using calibrated gas mixture from cylinder (2 ppmv). Acquisition time was 1 s; (**b**) retrieved concentration as nitrogen, gas from cylinder (2 ppmv of CH_4_) and lab air were flown through the cell (magenta—1 Hz data; black—5 s moving average); (**c**) 4f/1f WMS spectra at 1574.5 nm of lab air (pink) and calibrated gas mixture from cylinder (5 ppmv of hydrogen sulfide; black). Acquisition time was 30 s.

During the experiments, we found that the long term stability of the system is limited by three factors: stability of the laser wavelength, optical fringes, and fluctuations of the baseline. These issues can be addressed by applying a wavelength scanning and with full WMS spectrum being recorded and analyzed. In a scanned mode, 100 points were collected during each scan at 100 Hz (10 ms per point) and several consecutive scans could be acquired and averaged before spectral analysis. As a result, acquisition times in the order of 10 to 30 s must be used in order to obtain detection limits estimated earlier in a line-locked mode. Signal post processing included baseline and peak fitting (using linear function and a second order polynomial) for both measurement and calibration paths. Figure 6 shows sample spectra recorded using calibrated gas mixtures from cylinders: a single 2*f*/1*f* WMS spectrum of 2 ppmv of methane (acquired within 1 s) is demonstrated in Figure 6a; retrieved methane concentration as multi-pass cell was filled with different gas mixtures is shown in Figure 6b; Figure 6c shows 4*f*/1*f* WMS signal when 5 ppmv of H_2_S was flown through a multi pass cell. Detection limit of H_2_S at single ppmv level can be obtained after averaging 30 scans (acquisition time of 30 s), which is consistent with Allan deviation analysis (that was performed in a line-locked mode).

### 3.2. Linearity

The linearity of the technique was experimentally analyzed by measuring the signal for different samples of methane and hydrogen sulfide. Verification of linearity for these two molecules is particularly important: for methane, because expected concentrations will result in the highest peak absorption among all four gasses (up to few percent); for hydrogen sulfide, because of large difference between concentrations that are expected to be measured (up to ~20 ppmv) and concentration in a reference cell (pure H_2_S at 50 mm corresponds to 1667 ppmv at 30 m).

**Figure 7:**
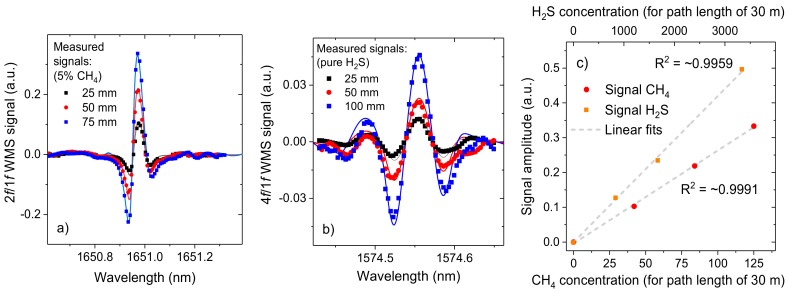
(**a**) 2*f*/1*f* WMS-based sensor signals for three methane samples. Measured concentrations correspond to 42, 83 and 125 ppmv for the path length of 30 m. Good agreement between measured signals and numerical model based on HITRAN database is obtained (model is plotted using solid line); (**b**) similar 4*f*/1*f* WMS measurement for three samples with pure H_2_S (corresponding concentrations at 30 m are 833, 1667, and 3333 ppmv); (**c**) linearity of the sensor system for methane and hydrogen sulfide detection.

For experimental verification of the linearity, a multi-pass cell in the setup was replaced with an appropriate glass cell. First, three samples with 5% of methane balanced with nitrogen at 740 Torr, enclosed in cells with optical path lengths of 25, 50, and 75 mm were used. For an optical path length of 30 m these concentration correspond to 42, 83, and 125 ppmv of methane. Figure 7a plots the obtained 2*f*/1*f* WMS signals together with simulated spectra based on HITRAN database. A similar experiment was subsequently performed using three glass cells with pure H_2_S at 740 Torr, with optical path lengths of 25, 50, and 100 mm (correspond to 833, 1667, and 3333 ppmv at 30 m). Measured 4*f*/1*f* WMS spectra are shown in Figure 7b. Linear fitting for both gases is shown in Figure 7c. *R*^2^ values above 0.99 are obtained, which confirms the linearity of the sensor response. 

## 4. System Performance

**Figure 8:**
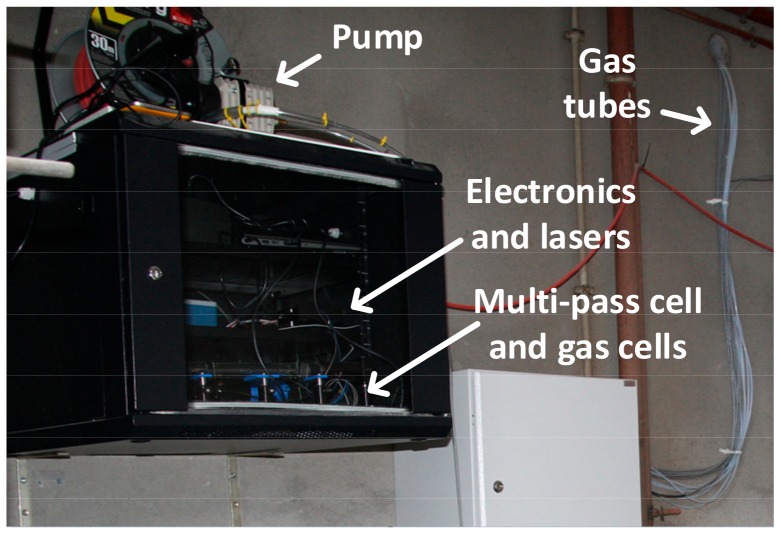
Picture of the sensor installed in the measurement site.

For its initial demonstration, the system was placed in a 19” rack cabinet (12U) and taken to a pig farm located in the Wielkopolska Region (in western Poland) where pigs of various ages are kept in different barn rooms. A set of teflon tubes was installed to deliver air samples from these rooms to the system that was installed in a boiler-room (shown in Figure 8). Acquisition times for each spectral range were set to 5 (CH_4_ detection), 15 (CO_2_ and H_2_S detection), and 10 s (NH_3_ detection). 

### 4.1. Stability Test 

**Figure 9:**
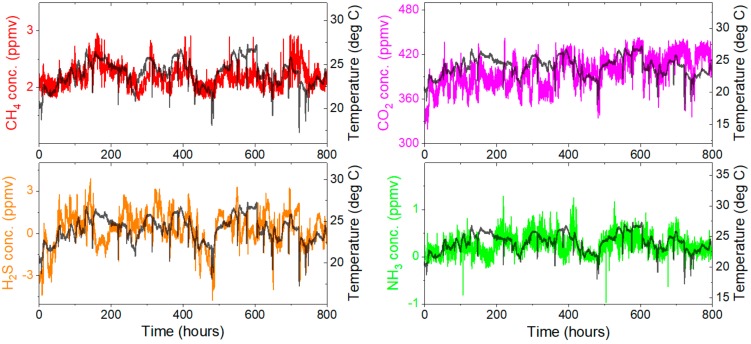
Retrieved concentrations of methane, carbon dioxide, hydrogen sulfide, and ammonia between 1 November 2017 and 4 December 2017 (“0” hour corresponds to the midnight between 31 October and 1 November). Ambient temperature is also plotted to show some correlation between recorded signals and ambient conditions (temperature inside instrument and multi-pass cell was also recorded and it was ~1.5 degrees higher than ambient temperature during the whole measurement).

Three main sources of uncertainties during field measurements can be expected, all being related to changes in ambient conditions (primarily temperature fluctuations). The first is due to temperature-induced changes in absorption line parameters, such as line strength and shape. This contribution can be estimated using numerical model and data from HITRAN database: depending on target transition and wavelength modulation amplitude the impact of temperature on measured amplitudes is between −0.12%/K and −0.15%/K (e.g., for methane, hydrogen sulfide and ammonia this corresponds to the change of the retrieved concentration of only tens of ppbv per Kelvin at most). The second source of uncertainty is related to changes in the path length. A multi-pass cell was constructed using stainless steel rods. Assuming thermal expansion coefficient of stainless steel is 17.3 × 10^−6^ 1/K the impact of temperature changes on measured signal is approximately 0.13%/K. Therefore, these two contributions (both being proportional to measured amplitude) should nearly cancel each other. The third source of uncertainty is due to optical fringes and baseline drifts. To estimate this contribution the system was installed and run with no air pump, with multi-pass cell filled with laboratory air and sealed. The sensor was running for over one month from 1 November to 4 December 2017, retrieving molecular concentrations and temperature inside the instrument. Recorded date sets are shown in Figure 9. Some correlation between ambient temperature and retrieved concentrations can be noticed. The presence of temperature-induced changes in all four measurements (where H_2_S and NH_3_ data sets have mean values ≈ 0) suggests that their origin is primarily in drifts of baseline and optical fringe. Observed fluctuations correspond to fractional absorptions of ~10^−4^ (CO_2_ measurement), ~2 × 10^−4^ (H_2_S and NH_3_) and ~5.5 × 10^−4^ (CH_4_). Small differences are primarily due to different wavelength modulation amplitudes and different harmonics being analyzed. 

### 4.2. Gas Emission Measurements 

**Figure 10:**
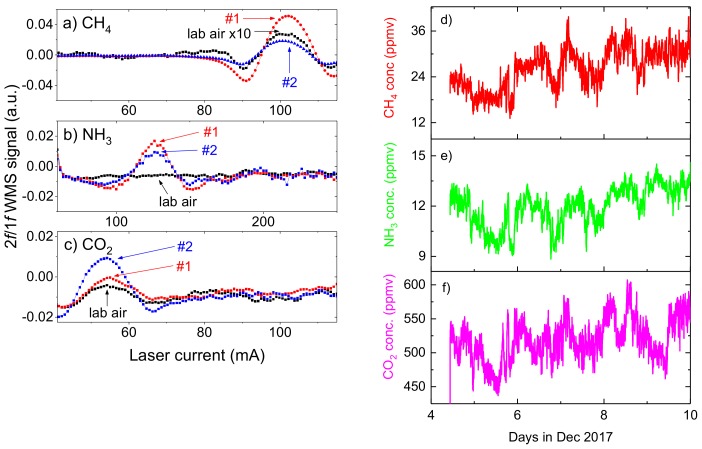
WMS spectra recorded at 1651 (**a**), 1531 (**b**), and 1574.5 (**c**) nm for three different air samples. Signals were recorded in August 2017 with initial version of the system. Retrieved concentrations are pig farm, room #1: CH_4_ = 38.12 ppmv, NH_3_ = 11.55 ppmv, CO_2_ = 583 ppmv; pig farm, room #2: CH_4_ = 14.58 ppmv, NH_3_ = 8.07 ppmv, CO_2_ = 1456 ppmv; laboratory air: CH_4_ = 1.89 ppmv, NH_3_ = no signal, CO_2_ = 395 ppmv; (**d**–**f**) concentrations of methane, ammonia, and carbon dioxide were recorded over one week in early December, 2017.

When a small pump was installed air was flown through the multi-pass cell (no pressure controllers were used) and samples from different rooms could be analyzed. Sample spectra are shown in Figure 10a–c, compared with signals recorded in laboratory (before the system was taken to the actual measurement site). Samples from two mechanically ventilated rooms were analyzed: 18 sows with suckling piglets (0–4 weeks old) were kept in room #1, while about 240 older piglets (5–10 weeks old) were kept in room #2. Concentration of methane in room #1 was found to be at ~38 ppmv, while ~15 ppmv was measured in room #2. Similarly, concentration of ammonia in room #1 was higher than in room #2 (~11.5 ppmv and ~8 ppmv, respectively). This is primarily because sows emit much larger amounts of methane and ammonia (as compared to piglets). Concentrations of carbon dioxide in room #1 and #2 were ~580 ppmv and ~1460 ppmv, respectively. This difference may reflect ventilation rate in both rooms, caused by the need to maintain higher temperatures in the room for leftover piglets compared to the room for sows with piglets. No signal from hydrogen sulfide was recorded. Obtained results were consistent with NH_3_, CO_2_, and H_2_S measurements taken with manually-operated MultiRAE Lite device.

Subsequently, the sensor was left for several days, analyzing air samples from one of the rooms in the farm with mid-size animals. Measured concentrations of methane, ammonia, and carbon dioxide are shown in Figure 10d–f. These are only preliminary results, but some periodicity can be observed in retrieved data sets. It is visible especially for ammonia measurement: concentration appears to be higher during the day and lower during the night. These changes seem not to be correlated with ambient temperature fluctuations and may be related e.g., to the activity of pigs [37]. 

## 5. Conclusions

In this paper, a transportable, laser-based system for sequential detection of hydrogen sulfide, methane, carbon dioxide, and ammonia was presented. Using near-infrared sources, wavelength modulation spectroscopy, and a Herriot-type multi-pass cell, sub-ppmv detection limits for CH_4_ and NH_3_ were obtained. Measuring concentrations of H_2_S was found to be the most challenging due to interference from CO_2_ lines located in the proximity of hydrogen sulfide transition. Especially since the instrument was designed to operate at atmospheric pressure and measured absorption features were relatively broad. This issue was addressed by application of WMS technique with signal detection at the fourth harmonic of the modulation frequency that enabled H_2_S sensing at single parts-per-million level with strongly reduced interference from adjacent CO_2_ transitions. This performance was obtained in a simple and robust configuration which does not require pressure controllers or external gas cylinders. Setup characterization in laboratory conditions was presented. Initial tests in a pig farm facility enabled analyzing system performance during field operation. Early results suggest that sensor system is stable enough to perform long-term measurements of CH_4_ and NH_3_ concentrations with accuracy below 1 ppmv. With this performance, we can use it to analyze how emission of methane and ammonia depends on various factors such as weather conditions, ventilation rate, animals activity, or farm maintenance schedule. 

Further work will be focused on improving the accuracy of hydrogen sulfide detection. Potential modifications may involve implementing more advanced signal processing algorithms that include e.g., temperature compensation of measured signals, or improving system alignment and optical coatings for optical fringes reduction. We are also working on methodology for verifying the results obtained from the measuring system at its assembly site. During long-term deployment, we will also analyze the impact of gas sampling lines on system performance and its accuracy. 

## Figures and Tables

**Table 1:** Details on chosen spectral regions, target transitions, and required detection limits

Spectral Region	Target Gas	Target Line Position and FWHM ^1^	Required Detection Limit (ppmv)	Fractional Absorption ^2^
1531 nm	NH_3_	6528.9 cm^−1^ and 24.29 GHz	1	3 × 10^−4^
1575 nm	CO_2_ H_2_S	6351.6 cm^−1^ and 7.06 GHz 6351.0 cm^−1^ and 10.57 GHz	100 2	1.71 × 10^−4^ 0.94 × 10^−4^
1651 nm	CH_4_	6057.1 cm^−1^ and 5.00 GHz	1	10.15 × 10^−4^

^1^ Full width at half maximum; ^2^ For required detection limit and path length of 30 m.
